# The first human clinical study for NMN has started in Japan

**DOI:** 10.1038/npjamd.2016.21

**Published:** 2016-10-27

**Authors:** Kazuo Tsubota

**Affiliations:** 1 Department of Ophthalmology, Keio University School of Medicine, Tokyo, Japan

In the recently published review in *npj Aging and Mechanisms of Disease,* Imai and Guarente^1^ have provided a stimulating review about the role of nicotinamide adenine dinucleotide (NAD) in aging. The best known intervention against aging is calorie restriction, which is now recognized to be at the center of aging research. In 2000, Imai *et al*.^2^ published a seminal paper in *Nature* entitled ‘Transcriptional silencing and longevity protein Sir2 is an NAD-dependent histone deacetylase’. In this paper, they proposed a possible mechanism underlying the benefit of calorie restriction—Sir2 upregulator. Since then, Sir2 family proteins, now called sirtuins, and NAD have attracted researchers’ significant attention for the intervention to promote longevity. The coupling of sirtuin activity and NAD breakdown is a unique mechanism, and the authors portray it with the apt idiom ‘it takes two to tango’. Indeed, as in tango, both sirtuins and NAD are necessary for healthy aging and longevity. When NAD levels decrease with aging, the NAD/sirtuin ‘tango’ falters.

The intervention against aging is now an emerging application. Last year, Kaeberlein *et al.*^3^ described eight possible interventions as disease preventive methods. The interventions include: dietary restriction, exercise, mechanistic target of rapamycin (mTOR) inhibitors, metformin and acarbose, NAD precursors and sirtuin activators, modifiers of senescence and telomore dysfunction, hormonal and circulating factors, and mitochondria-targeted therapeutics. Among them, dietary restriction (calorie restriction) and exercise appear to be the realistic interventions, but both requires modest lifestyle modification which may be difficult for some individuals to implement. mTOR inbibitors, as well as telomere modifiers, can be good practical targets, but they are still under investigation. Metformin and acarbose are now considered to be possible anti-aging drugs which are ready for a clinical trial in the US.^4^ Researchers have already proven that the diabetes drug metformin extends the life span of animals,^5^ and the Food and Drug Administration in the US has now given a go for a trial to see if the same effects can be replicated in humans. Among these practical intervention strategies, NAD precursors and sirtuin activators are also attracting significant attention owing to the vast amount of data accumulated in the last 16 years since the discovery of the NAD and sirtuin interaction. Although NAD itself is difficult to administer directly to humans, its precursors—nicotinamide riboside (NR) and nicotinamide mononucleotide (NMN)—are promising natural compounds with which to augment NAD levels in the cells and the body. Both NR and NMN have been shown to be beneficial to ameliorate complications in glucose metabolism, cardiovascular and neural functions, and stem cell maintenance, and even promote longevity in some models. For NR, several clinical studies have already been ongoing in the US and Europe.

Although NMN is already available on the market, the safety and effect on human physiology remain unknown. Recently, an international collaborative team between Keio University School of Medicine in Tokyo and Washington University School of Medicine in St Louis has started the phase I human clinical study for NMN.^6^ At Keio, Hiroshi Itoh, Professor of Endocrinology, Metabolism, and Nephrology and his team are leading the study. Shin-ichiro Imai, Professor of Developmental Biology, is involved in the study at the US side. The aim of this initial study is to assess the safety and the bioavailability of NMN in humans. Ten healthy volunteers will be recruited, and the safety and the time course of blood NMN concentrations will be evaluated. From this human phase I clinical study, we will be able to learn how NMN behaves in the human body. It should be particularly emphasized that this phase I study is not planned for a pharmaceutical development, but for a nutraceutical development, although both NMN and NR can be potentially developed as pharmaceutical drugs. Drug development for disease treatment or prevention takes considerable amounts of time and money due to rigorous regulations and strict clinical trial adherence. The advantage of developing NMN as a nutraceutical could possibly save time and costs, accelerating future clinical applications. In this regard, this clinical study on NMN aims to provide a critical scientific foundation for the rigorous development of NMN as an anti-aging nutraceutical.

The clinical application of basic aging research is now on the horizon worldwide. The intervention with natural compounds, such as NMN or NR, can be a promising strategy, and enthusiasm is now growing in this field of geroscience. An important aim of *npj Aging and Mechanisms of Disease* is to cover such an emerging clinical field of aging research, which is increasingly relevant for heavily aging societies around the world, including Japan.
